# Healthcare and treatment experiences among people diagnosed with HIV before and after a province-wide treatment as prevention initiative in British Columbia, Canada

**DOI:** 10.1186/s12889-022-13415-2

**Published:** 2022-05-21

**Authors:** Tessa Tattersall, Clara Tam, David Moore, Tim Wesseling, Sean Grieve, Lu Wang, Nic Bacani, Julio S. G. Montaner, Robert S. Hogg, Rolando Barrios, Kate Salters

**Affiliations:** 1 British Columbia Centre for Excellence in HIV/AIDS, 608-1081 Burrard Street, Vancouver, BC V6Z1Y6 Canada; 2 Faculty of Medicine, University of British Columbia, Vancouver, BC Canada; 3 Faculty of Health Sciences, Simon Fraser University, Burnaby, BC Canada

**Keywords:** HIV/AIDS, Patient care experiences, ART initiation, Virologic suppression, Treatment as Prevention (TasP)

## Abstract

**Introduction:**

In 2010, the Canadian province of British Columbia (BC) initiated the Seek and Treat for Optimal Prevention of HIV/AIDS (STOP HIV/AIDS) program to improve HIV testing, linkage to care, and treatment uptake, thereby operationalizing the HIV Treatment as Prevention (TasP) framework at the population-level. In this analysis, we evaluated self-reported HIV care experiences and therapeutic outcomes among people diagnosed with HIV prior to and after implementation of this provincial program.

**Methods:**

A cross-sectional analysis was performed on the baseline data of a cohort of people living with HIV (PLWH) (19 years and older) in the province of BC sampled from July 2016 to September 2018. All participants consented to linking their survey data to the provincial HIV treatment registry. Individuals diagnosed with HIV from January 1 2000—December 31 2009 were classified as pre-intervention and those diagnosed January 1 2010—December 31 2018 as post-intervention cohorts. Bivariate analyses were run using Chi-square and Wilcoxon Rank Sum tests. Cox proportional hazards regression model demonstrates time to antiretroviral therapy (ART) initiation (from HIV baseline) and virological suppression (2 consecutive plasma viral load measurements < 200 copies/ml).

**Results:**

Of the 325 participants included in this analysis, 198 (61%) were diagnosed with HIV in the pre-intervention era and 127 (39%) in the post-intervention era. A higher proportion of participants in post-intervention era were diagnosed at walk-in clinics (45% vs. 39%) and hospitals (21% vs. 11%) (vs pre-intervention) (*p* = 0.042). Post-intervention participants had initiated ART with less advanced HIV disease (CD4 count 410 vs. 270 cells/ul; p = 0.001) and were less likely to experience treatment interruptions at any point in the 5 years after HIV diagnosis (17% vs. 48%; p < 0.001). The post-intervention cohort had significantly more timely ART initiation (aHR: 5.97, 95%CI 4.47, 7.97) and virologic suppression (aHR: 2.03, 95%CI 1.58, 2.60) following diagnosis, after controlling for confounders.

**Conclusions:**

We found favourable treatment experiences and more timely ART initiation and virologic suppression after a targeted TasP provincial program. Our results illustrate the importance of accessible low-barrier HIV testing and treatment in tackling the HIV epidemic.

## Introduction

There is overwhelming evidence demonstrating the impact of early testing and treatment for HIV on improved health and longevity among people living with HIV (PLWH) [1, 2]. Importantly, research has continued to demonstrate the larger impact of expanded treatment for HIV, including the virtual elimination of risk of onward HIV transmission among PLWH who immediately start and are sustained on ART [3, 4]. In practice, this concept known as Treatment as Prevention (TasP), necessitates the enhanced detection of cases of HIV and the universal provision of antiretroviral therapy (ART) for all eligible PLWH, irrespective of disease progression [1]. In British Columbia (BC), Canada, the concept of TasP was first championed in 2006 and a clear case was made for a pilot program in order to operationalize these principles and evaluate the impact on clinical and therapeutic outcomes [2].

On February 4, 2010, the BC Ministry of Health in partnership with the BC Centre for Excellence in HIV/AIDS (BC-CfE) launched the Seek and Treat for Optimal Prevention of HIV/AIDS (STOP HIV/AIDS) pilot program [5] in two urban cities (Vancouver and Prince George). In concert with expanded treatment guidelines that supported universal access to treatment across the province, the STOP HIV/AIDS program was a product of galvanized efforts to improve HIV testing and support linkage and retention in HIV care with the aim of improving the proportion of virologically suppressed PLWH, thereby reducing risk of HIV transmission [1, 6, 7]. This initiative, in partnership with regional health authorities, provided training for service providers, facilitated routine offers of HIV testing in all healthcare settings, and provided assistance for outreach nursing teams to support engagement and retention on ART. Acknowledging the pervasive barriers to care for under-served populations, the STOP HIV/AIDS initiative attempted to address health inequities among PLWH who are most structurally and socially marginalized in BC by applying an integrated model of HIV care which incorporates supportive services for concurrent health and psychosocial issues to optimize HIV care engagement [8, 9]. Population-level improvements in HIV clinical outcomes of the STOP HIV/AIDS initiative became quickly apparent, and therefore in 2012, the Ministry of Health agreed to expand the project on a province-wide basis, which launched on April 1 2013 [7, 10–12]. Moreover, in 2011, the first ‘Treatment as Prevention’ workshop was hosted by the BC-CfE and co-hosted by the International AIDS Society, Joint United Nations Program on HIV/AIDS, World Health Organization, and the National Institute on Drug Abuse; following endorsement of the TasP principles at an international level.

While success of the project in improving HIV testing, morbidity, mortality and transmission outcomes has been established, the research to date has focused primarily on clinical and therapeutic outcomes [7, 10, 11, 13]. To the best of our knowledge, there has been no evaluation of self-reported patient healthcare and treatment experiences before and after this province-wide initiative. As such, it is important to investigate both health outcomes and self-reported health care experiences because, particularly among under-served populations, such as women, youth, and people who use injection drugs, there are still disparities in clinical outcomes among PLWH [14]. Moreover, this study complements previous clinical evaluations and provides a necessary look at patient experiences around the time of their HIV diagnosis. We therefore undertook the present study to compare experiences of individuals diagnosed with HIV prior to and during the STOP HIV/AIDS program implementation in order to determine the effectiveness of the program in supporting PLWH.

## Methods

Beginning in January 2016 and ending in September 2018, our study team enrolled PLWH in a cohort known as the STOP HIV/AIDS Program Evaluation (SHAPE) study. The objectives of the SHAPE study were to evaluate the STOP program and to determine the HIV health care experiences and associated health care needs, beliefs, and behaviours of PLWH in BC, and the study has been described at length previously [15, 16].

### Study population, recruitment, and procedures

Eligibility for the SHAPE study includes people living with HIV in BC who were 19 or older as verified in the BC-CfE Drug Treatment Program (DTP) registry, able to complete the survey in English, and able to provide informed consent. Recruitment targets were developed based on the proportion of key sociodemographic characteristics of PLWH in BC, using estimates derived from DTP in order to ensure representativeness of the study population. The DTP provides longitudinal clinical (i.e. viral load, CD4 cell count), laboratory (i.e. resistance testing, phylogenetic monitoring), therapeutic (i.e. ART regimen), and adherence data (i.e. pharmacy refill) for all individuals who receive ART in the province. SHAPE study participants were recruited using purposive sampling in order to ensure representation based on age, gender, HIV risk group, and health authority of residence. Participants were recruited through posting advertisements on Facebook, Craigslist, Grindr, and Scruff as well as through posters and postcards via existing and new partnerships with clinics, pharmacies, and AIDS Service Organizations. This analysis concerns the baseline survey collected from January 1, 2016 to September 1, 2018. The one-hour surveys were conducted either independently online or were administered by a Peer Research Associate (PRA), who are trained research team members with living experience with HIV either in-person or over the phone. A $30 cash honorarium was given to each participant for participation in the baseline study. As part of the enrolment procedures, participants were asked to provide consent to link their survey responses to longitudinal clinical, laboratory, and pharmacy refill data from the provincial DTP, which was verified prior to the enrolment in order to ensure linkage to clinical data for all participants. This linkage was completed through matching personal health numbers, name and date of birth. Inclusion criteria for this analysis included participants who completed the baseline survey, were linked to the DTP clinical registry, and had an HIV diagnosis date of January 1, 2000 or later, and were ART-naïve at the start of first ART dispensation.

The protocol was approved by the Research Ethics Board of the University of BC/Providence Health Care (ID Number H15-01807). Written informed consent was obtained from each participant for in-person and phone interviews, and electronically for online surveys. All identifying information was removed from the analysis and cells with counts of less than 5 were suppressed.

## Measures

The exposure of interest was the date of HIV diagnosis which, for 178 out of 325 participants who had a DTP diagnosis date, was derived by the mid-point estimation between self-reported diagnosis date and DTP registry date. For the remaining 147 participants, DTP clinical registry data was used to derive an approximate HIV diagnosis date from the earliest date of first AIDS-defining illness, first detectable viral load, or first ART date (whichever occurred first). HIV diagnosis date was dichotomized to before and after the implementation of the provincial STOP HIV/AIDS initiative (2010 onwards). It is important to note that there was variation in the time of implementation between health authorities, as noted earlier, however, it was during this roll-out of the associated HIV therapeutic guideline changes and pilot phases of BC HIV prevention and care initiatives, that helped establish increased funding and infrastructure that was later implemented province-wide [10]. The year 2010 onward signalled a significant shift provincially in HIV testing measures and how HIV was treated across the province, not just in terms of regimens, but where and when treatment was offered. As such, it was decided amongst the co-authors, many of whom were provincial leaders with the STOP program, that the years prior to 2010 would constitute the pre-intervention era, while 2010 and later would be the post-intervention era.

Explanatory variables of interest were populated using baseline survey data from eligible SHAPE participants. This included relevant socio-demographic variables including: gender identity (men vs women vs transgender/other), sexual orientation (straight vs gay vs other), health authority of residence, highest level of education (< high school vs ≥ high school), employed at the time of baseline survey (yes vs no), ever incarcerated as an adult (yes vs no), ever homeless (yes vs no), stable housing (strongly agree vs somewhat agree vs neutral vs somewhat disagree vs strongly disagree), and indication of food security using questions from the partial Household Food Security Survey module of the Canadian Community Health Survey (CCHS) [17] (sufficient vs insufficient). We also assessed health background information including: ever diagnosed with hepatitis B virus (HBV) (yes vs no), ever diagnosed with hepatitis C virus (HCV) (yes vs no), history of drug use (cocaine, crystal methamphetamine, heroin), history of ever injecting non-prescription drugs (yes vs no), indication of depressive symptoms using the CES-D 10 scale [18, 19] (non-significant depressive symptoms < 10 vs significant depressive symptoms ≥ 10), and CD4 cell count (cells/mm3) at the time of HIV diagnosis and at the time of ART initiation. Self-reported experiences of health care at the time of HIV diagnosis were also collected via the SHAPE baseline survey. These included questions on who diagnosed you with HIV (family doctor vs walk-in clinic vs hospital vs outreach nurse vs self-test vs other), guidance on initiating ART (start right away vs personal decision vs delay starting ART), perceived easy access to HIV-related care after diagnosis (yes vs somewhat vs no), self-reported difficulties accessing care, desire to start ART immediately (yes vs no), most important reason for starting ART, resources that were desired by participants but not used at the time of HIV diagnosis, and treatment interruptions experienced (≥ 90 days gap in ART dispensation) at any point in the five years following diagnosis.

Our primary clinical outcome measures of interest from the DTP clinical registry included time from HIV diagnosis to date of first ART dispensation, and the time from ART initiation to virological suppression (defined as 2 consecutive plasma viral load (pVL) measurements < 200 copies/ml).

### Statistical analysis

We used Chi-square and Wilcoxon Rank Sum tests to compare the distribution of sociodemographic, experiential, and engagement in care variables with the exposure of prior to and during the provincial STOP HIV/AIDS program. Univariable and multivariable Cox proportional hazards regression models were used to assess both the time to ART initiation from HIV diagnosis date and the time to virological suppression from ART initiation date between participants diagnosed with HIV prior to and during the STOP HIV/AIDS era. The multivariable models adjusted for confounding variables of age, gender, history of ever using injection drugs, and sexual orientation. Kaplan–Meier curves displayed both the difference in time to ART initiation and time to virological suppression between prior to and during STOP HIV/AIDS era groups.

## Results

Of 644 SHAPE participants, three were excluded due to incomplete baseline surveys, 256 were excluded due to having an HIV diagnosis date prior to January 1, 2000, 53 were excluded due to initiating ART outside of BC, and seven were excluded because ART-naïve status at the time of entry into the DTP could not be confirmed. After applying exclusion criteria, we had 325 participants that were included in this analytic sample.

### Demographics and health status

Of the 325 (50.5% of 644) participants from the SHAPE baseline survey eligible for inclusion in this analysis, 198 (61%) were diagnosed with HIV in the pre-STOP HIV/AIDS era and 127 (39%) were diagnosed during the post-STOP HIV/AIDS era. The median age of individuals at diagnosis in the analytic sample was 37 years old (Q1-Q3: 30–46), 26.2% were women, 72.3% were men, < 2% were transgender or non-binary individuals, 38.8% identified as gay, 48.3% lived within the Vancouver Coastal Health Authority, and 64.2% had insufficient food security (see Table 1).

**Table 1:** Characterizing sociodemographic profiles and treatment experiences among people living with HIV before and after the implementation of the provincial STOP HIV/AIDS program in British Columbia, Canada

Variable	Overall (*n* = 325) n (%)	Pre- STOP HIV/AIDS (*n* = 198) n (%)	Post-STOP HIV/AIDS (*n* = 127) n (%)	*P*-Value
Gender identity				**0.004**
Man	235 (72.3)	131 (66.2)	104 (81.9)	
Woman	85 (26.2)	64 (32.3)	21 (16.5)	
Transgender and non-binary individuals^a^	5 (1.5)	< 5	< 5	
Sexual orientation				**0.039**
Straight	146 (44.9)	100 (50.5)	46 (36.2)	
Gay	126 (38.8)	68 (34.3)	58 (45.7)	
Other	53 (16.3)	30 (15.2)	23 (18.1)	
Health Authority				0.826
Interior Health	20 (6.2)	13 (6.6)	7 (5.5)	
Fraser Health	65 (20.0)	37 (18.7)	28 (22.0)	
Vancouver Coastal Health	157 (48.3)	98 (49.5)	59 (46.5)	
Vancouver Island Health	35 (10.8)	23 (11.6)	12 (9.4)	
Northern Health	48 (14.8)	27 (13.6)	21 (16.5)	
Education				**0.001**
Incomplete high school	102 (31.4)	76 (38.4)	26 (20.5)	
High school or greater	223 (68.6)	122 (61.6)	101 (79.5)	
Employed	120 (36.9)	69 (34.8)	51 (40.2)	0.333
Ever incarcerated as adult	134 (41.2)	95 (48.0)	39 (30.7)	**0.002**
Ever homeless				0.103
No	143 (44.0)	80 (40.4)	63 (49.6)	
Yes, currently or previously	182 (56.0)	118 (59.6)	64 (50.4)	
Stable housing				0.852
Strongly agree	154 (47.4)	93 (47.0)	61 (48.0)	
Somewhat agree	92 (28.3)	59 (29.8)	33 (26.0)	
Neutral	32 (9.8)	19 (9.6)	13 (10.2)	
Somewhat disagree	22 (6.8)	14 (7.1)	8 (6.3)	
Strongly disagree	25 (7.7)	13 (6.6)	12 (9.4)	
Food security (CCHS)^b^				0.355
Sufficient	116 (35.8)	67 (33.8)	49 (38.9)	
Insufficient	208 (64.2)	131 (66.2)	77 (61.1)	
Ever diagnosed with hepatitis B	40 (12.3)	32 (16.2)	8 (6.3)	**0.008**
Ever diagnosed with hepatitis C	118 (36.4)	95 (48.2)	23 (18.1)	** < 0.001**
Ever used cocaine	226 (69.5)	141 (71.2)	85 (66.9)	0.413
Ever used crystal methamphetamine	166 (51.7)	95 (48.5)	71 (56.8)	0.145
Ever used heroin	113 (34.8)	79 (39.9)	34 (26.8)	**0.015**
Ever injected non-prescription drugs	151 (46.5)	105 (53.0)	46 (36.2)	**0.003**
Depression (CES-D 10)^c^				0.415
Non-significant depressive symptoms	141 (47.5)	87 (49.4)	54 (44.6)	
Significant depressive symptoms	156 (52.5)	89 (50.6)	67 (55.4)	
Location of HIV diagnosis				**0.042**
Family doctor	90 (27.7)	64 (32.3)	26 (20.5)	
Walk-in clinic	135 (41.5)	78 (39.4)	57 (44.9)	
Hospital	48 (14.8)	22 (11.1)	26 (20.5)	
Outreach nurse	31 (9.5)	20 (10.1)	11 (8.7)	
Self-test	< 5	< 5	< 5	
Other	20 (6.2)	14 (7.1)	6 (4.7)	
Advice provided about ART initiation from health care provider when diagnosed (*n* = 297)				**0.008**
Start ART right away	158 (48.6)	84 (42.4)	74 (58.3)	
Up to me	61 (18.8)	41 (20.7)	20 (15.7)	
Delay starting ART	78 (32.6)	60 (36.9)	18 (26.0)	
Easy to access HIV-related care after HIV diagnosis?				0.233
Yes	264 (81.2)	155 (78.3)	109 (85.8)	
Somewhat	50 (15.4)	35 (17.7)	15 (11.8)	
No	11 (3.4)	8 (4.0)	< 5	
Difficulties faced when accessing HIV-related care:				
Services not available	11 (3.4)	5 (2.5)	6 (4.7)	0.350
Services too far	20 (6.2)	13 (6.6)	7 (5.5)	0.700
Wait time	14 (4.3)	7 (3.5)	7 (5.5)	0.392
Didn’t know where to go	9 (2.8)	8 (4.0)	< 5	0.096
Didn’t know who to talk to	23 (7.1)	19 (9.6)	< 5	**0.027**
Indicated a personal desire to start ART immediately after diagnosis	173 (53.4)	79 (40.1)	94 (74.0)	** < 0.001**
Most important reason for starting ART (*n* = 324)				0.052
Doctor advised me to and explained why	127 (39.2)	84 (42.6)	43 (33.9)	
I wanted to stay healthy	89 (27.5)	48 (24.4)	41 (32.3)	
I was feeling sick	25 (7.7)	20 (10.2)	5 (3.9)	
In hospital and had to	19 (5.9)	9 (4.6)	10 (7.9)	
Concerned about transmitting HIV to partner	14 (4.3)	5 (2.5)	9 (7.1)	
Doctor advised me to but didn’t explain why	12 (3.7)	9 (4.6)	< 5	
Other HIV positive people were on ART	< 5	< 5	< 5	
I had another condition/infection	9 (2.8)	5 (2.5)	< 5	
Participating in a research study	< 5	< 5	< 5	
I was pregnant	6 (1.9)	< 5	< 5	
Other	11 (3.4)	6 (3.0)	5 (3.9)	
Resource desired, but not used:				
Discussion with health care providers	9 (2.8)	5 (2.5)	< 5	0.739
Discussion with family and friends	28 (8.6)	15 (7.6)	13 (10.2)	0.381
Discussion with friends living with HIV	18 (5.5)	7 (3.5)	11 (8.7)	**0.045**
Community organization information	16 (4.9)	9 (4.5)	7 (5.5)	0.672
Online resources	18 (5.5)	9 (4.5)	9 (7.1)	0.312
Pamphlets	17 (5.2)	9 (4.5)	8 (6.3)	0.469
Other	9 (2.8)	6 (3.0)	< 5	0.999
At least one treatment interruption event within five years from ART initiation	117 (36.0)	95 (48.0)	22 (17.3)	** < 0.001**
V**ariable**	**Median (Q1-Q3)**	**Median (Q1-Q3)**	**Median (Q1-Q3)**	***P*-Value**
Age at diagnosis (years)	37 (30–46)	37 (31–44)	38 (29–47)	0.258
CD4 count at first ART (*n* = 324, cells/ul)	310 (180–497)	270 (170–430)	410 (220–620)	**0.001**

Missing values excluded from the table. Bolded text indicates significant results at *P* < 0.05

Cells with values < 5 are suppressed

^a^Inclusive of transgender women, transgender men, and non-binary participants

^b^Canada. Office of Nutrition Policy and Promotion. *Canadian community health survey. Cycle 2.2, Nutrition (2004): income-related household food security in Canada.* (Office of Nutrition Policy and Promotion, Health Canada, 2007); includes adapted version

^c^Zhang, W. et al*.* Validating a shortened depression scale (10 item CES-D) among HIV-Positive people in British Columbia, Canada. *PLoS One* **7**, (2012)

Participants diagnosed with HIV during the STOP HIV/AIDS era were more frequently men (81.9% vs. 66.2%; *p* = 0.004), identified as gay (45.7% vs. 34.3%; *p* = 0.039), and had completed high-school or greater (79.5% vs. 61.6%; *p* = 0.001) in comparison to those diagnosed prior to the implementation of the provincial program. There was no significant difference between participants in the pre- vs post-intervention eras (median age of 37 and 38, respectively, *p* = 0.258). PLWH diagnosed during the post-intervention era were less likely to report a history of incarceration as an adult versus PLWH diagnosed prior to the provincial initiative (30.7% vs 48.0%; *p* = 0.002). History of homelessness was prevalent overall, with 59.6% and 50.4% reported being homeless currently or previously, prior to and during the post-intervention era, respectively, although there was no statistical difference between the two groups (*p* = 0.103).

Coinfection of HCV (18.1% vs. 48.2%; *p* < 0.001), HBV (6.3% vs. 16.2%; *p* = 0.008), self-reported ever using heroin (26.8% vs. 39.9%; *p* = 0.015), and lifetime injection drug use (36.2% vs. 53.0%; *p* = 0.003) were less frequent among post-intervention participants. Indication of significant depressive symptoms (CES-D 10 scale scores of ≥ 10 [18, 19]) remained highly prevalent across eras (pre-STOP HIV/AIDS: 50.6%; post-STOP HIV/AIDS: 55.4%; *p* = 0.415).

### HIV experiences of care

**Fig. 1:**
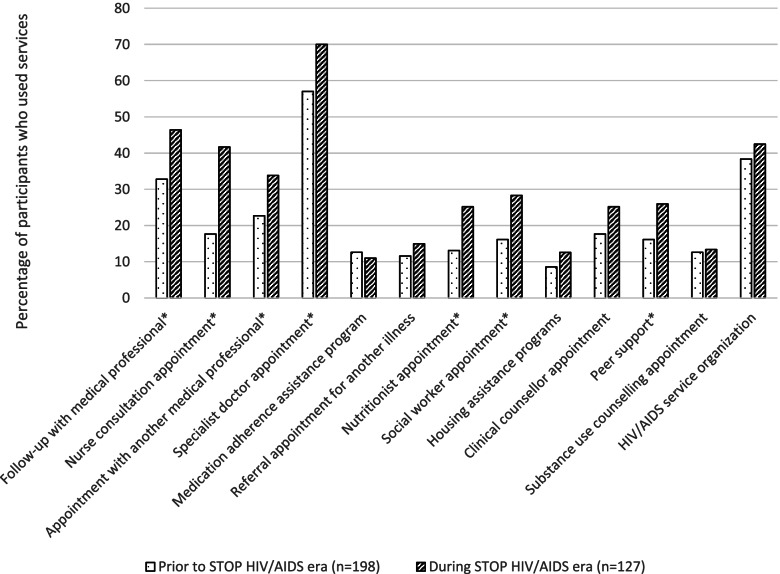
Use of HIV Care and Support Services following HIV diagnosis prior to and during the STOP HIV/AIDS program implementation. * = statistically significant difference (*p* ≤ 0.05)

**Fig. 2:**
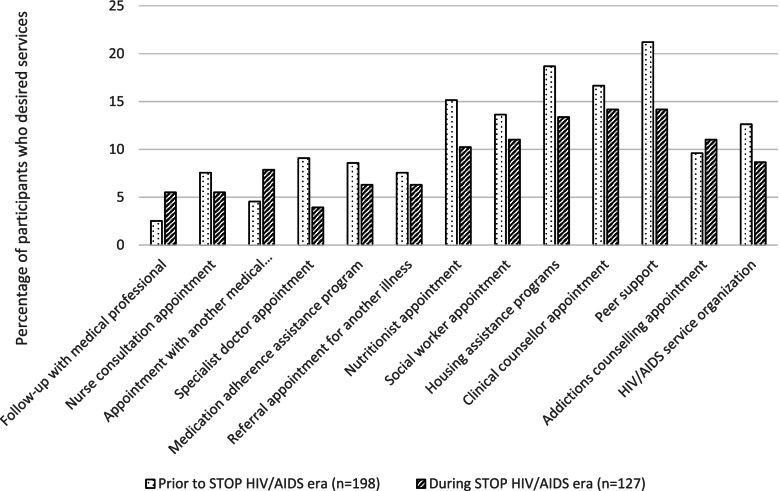
Desire for HIV Care and Support Services following HIV diagnosis prior to and during the STOP HIV/AIDS program implementation

There were important differences in the clinical experiences around HIV diagnoses reported in this analysis, with participants in the post-STOP era reporting fewer HIV diagnoses with a family doctor (20.5% vs 32.3%) and a greater proportion of HIV diagnoses with walk-in clinics (44.9% vs 39.4%) and hospitals (20.5% vs 11.1%) compared to PLWH diagnosed in the pre-STOP HIV/AIDS era (*p* = 0.042) (see Table 1). Participants diagnosed in the post-STOP HIV/AIDS era reported higher use of clinical services such as follow-up appointments with their diagnosing physician (46.5% vs 32.8%,%, *p* = 0.014), nurse consultations (41.7% vs 17.7%, *p* < 0.001), appointments with another medical professional (33.9% vs 22.7, *p* = 0.028), and visits with specialists (70.1% vs 57.1%, *p* = 0.018), than pre-STOP HIV/AIDS participants (see Fig. 1). Furthermore, the resources allocated in the regional health authorities as part of the STOP HIV/AIDS program for psycho-social supports led to increased utilization of nutritionist appointments (25.2% vs 13.1%, *p* = 0.006), social workers (28.3% vs 17.7%, *p* = 0.008), and peer support (26.0% vs 16.2%, *p* = 0.031), in the post-STOP HIV/AIDS era. Desire for HIV care and supportive services following HIV diagnosis did not differ significantly (*p* > 0.05) between participants in the two groups (see Fig. 2). However, participants diagnosed in the post-STOP HIV/AIDS era were less likely to report a desire for counselling (post-intervention 14% vs pre-intervention 17%), peer support (14% vs 21%), substance use counselling (11% vs 10%), medication adherence assistance programs (6% vs 9%), a nutritionist (10% vs 15%), a social worker (11% vs 14%), and housing assistance (13% vs 19%). This is a reflection of increased provision and availability of nursing, outreach, and psycho-social services, addressing much needed gaps in care identified in the cohort diagnosed with HIV prior to the STOP HIV/AIDS implementation.

### HIV treatment outcomes

Median CD4 count at time of ART initiation was significantly higher among post-STOP HIV/AIDS participants with 410 cells/ul (Q1-Q3: 220–620) compared with 270 (170–430) (*p* = 0.001). Additionally, there was a significantly lower proportion of participants with treatment interruptions reported within five years following ART initiation in the post-STOP HIV/AIDS era (during 17.3% vs prior 48.0%; *p* < 0.001) (see Table 1).

**Fig. 3:**
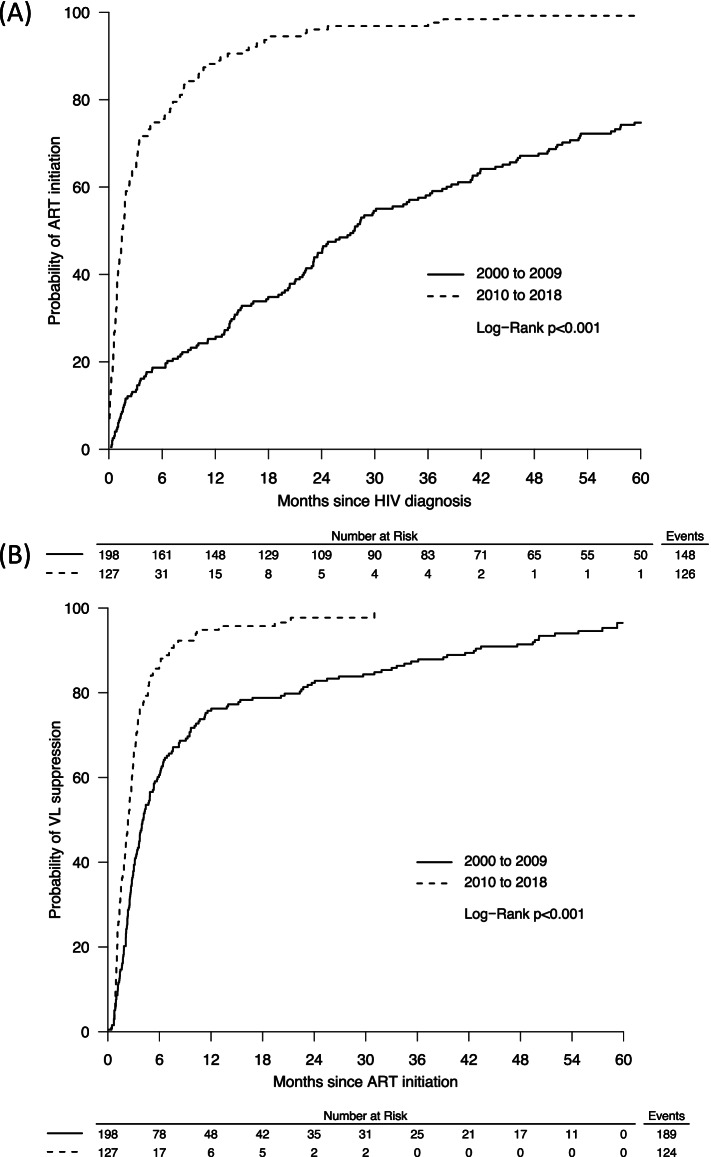
Kaplan Meier plots for time to ART initiation and virological suppression by STOP era

When adjusted for age, gender, sexual orientation, and history of injection drug use, STOP HIV/AIDS era participants were 5.97 times more likely to initiate ART than pre-STOP HIV/AIDS participants (adjusted hazards ratio [aHR] 5.96, 95% CI 4.47–7.97; *p* < 0.001) (see Table 2). Median time to ART initiation for STOP HIV/AIDS era participants was within 1.5 months of HIV diagnosis, compared to 27.5 months for participants prior to the provincial initiative (see Fig. 3A).

**Table 2:** Multivariable Cox regression analysis of time to ART initiation

Variable	Multivariable Cox Proportional Hazards Model				
	**ART initiation within study period**				
	**Yes vs No**				
	**aHR**	**95% CI**			***P*-value**
**Year of HIV diagnosis**					
2000 to 2009	REF				
2010 to 2018	5.966^*^	4.468		7.967	** < 0.001**
**Sexual Orientation**					
Straight	REF				
Gay	1.122	0.815		1.543	0.480
Other	0.977	0.671		1.424	0.904
**Ever Injected Substances**					
No	REF				
Yes	0.986	0.756		1.287	0.918
**Gender**					
Man	REF				
Woman	1.150	0.835		1.585	0.391
**Age at diagnosis (per 1 year increase)**	1.010	0.998	1.022		0.095

^*^Bolded text indicates significant results at *P* < 0.05

In the adjusted multivariable model, the STOP HIV/AIDS era participants were significantly more likely to reach viral suppression than prior to STOP HIV/AIDS era participants (aHR 2.03, 95% CI 1.58–2.60; *p* < 0.001) (see Table 3). Median time to virological suppression for STOP HIV/AIDS era participants was 2.3 months versus 4 months for prior to STOP HIV/AIDS participants (see Fig. 3B).

**Table 3:** Multivariable Cox regression analysis of time to virological suppression

Variable	Multivariable Cox Proportional Hazards Model			
	**VL suppression within study period**			
	**Yes vs No**			
	**aHR**	**95% CI**		**P-value**
**Year of HIV diagnosis**				
2000 to 2009	REF			
2010 to 2018	2.027^*^	1.583	2.595	** < 0.001**
**Sexual Orientation**				
Straight	REF			
Gay	1.669^*^	1.266	2.200	** < 0.001**
Other	1.233	0.884	1.719	0.217
**Ever Injected Substances**				
No	REF			
Yes	0.948	0.748	1.203	0.663
**Gender**				
Man	REF			
Woman	0.909	0.681	1.214	0.518
**Age at diagnosis (per 1 year increase)**	1.007	0.995	1.018	0.254

^*^Bolded text indicates significant results at *P* < 0.05

## Discussion

This is the first study, to our knowledge, assessing both patient-level experiences and timeliness of clinical outcomes made before and after the implementation of a TasP strategy from 2010 onwards. This study includes a cohort of diverse and socio-demographically representative PLWH engaged in care across the province of BC, with proportional representation by age, gender, HIV risk group, and geography [16], and includes major lessons and important considerations for future TasP implementation programs that aim to optimize testing and treatment for all PLWH.

Our study found that time to ART initiation and virologic suppression has significantly improved after the implementation of a province-wide Treatment as Prevention initiative. There was a nearly 6 times higher likelihood of timely ART initiation among participants diagnosed in the post-STOP HIV/AIDS era, which included the implementation of low-barrier testing and treatment programs. Our observations of less prevalent treatment interruption events in the era of STOP HIV/AIDS corroborates findings of sustained improvements in HIV care engagement [6]. These findings corroborate the clinical and therapeutic progress seen in the proportion of DTP enrollees on ART and virologically suppressed following TasP implementation [13].

Notably, in addition to these therapeutic successes, our study suggests that the TasP implementation program increased the use of clinical, psychosocial, and socioeconomic services at the time of HIV diagnosis. It is known that supportive services that address subsistence needs (i.e. food, shelter) and encourage social enablers facilitate HIV care engagement, reduced treatment interruptions, and improve the wellbeing of PLWH [20–23]. Our observations of increased use of peer support and social services can be attributed in part to their increased availability in the era of the STOP HIV/AIDS initiative [10]. Furthering the provision of supportive services within HIV care delivery is desired and will improve the wellbeing of PLWH and engagement in ART.

Location of HIV diagnosis and linkage to care has also changed with the implementation of the TasP initiative. The impact of offering low-barrier, point-of-care testing for HIV is reflected in our study results, as a greater proportion of people were diagnosed in a hospital, outreach or walk-in setting in the post-implementation era. Although point-of-care testing contributes to a small proportion of total true positive tests in BC [24], likely the routine offer of HIV testing in health care settings may have improved screening for HIV as suggested by previous literature [25, 26]. PLWH diagnosed during the post-STOP HIV/AIDS era were more likely to report their physician encouraged immediate initiation of ART compared to PLWH diagnosed in the pre-implementation era, which may also reflect updated treatment guidelines. However, not all healthcare providers in our analysis advised to start ART immediately, indicating that there are still opportunities to advance the adoption of TasP in clinical practice, suggesting a potential role for clinical education and training. Our results demonstrating the encouragement from physicians for timely ART initiation are noteworthy as timely diagnosis and treatment uptake are associated with reduced HIV transmission [13, 27–29] and reduced HIV-related morbidity and mortality [2, 27, 30–32]. These findings are justification for the success of the strategies implemented in the STOP HIV/AIDS era to improve screening of HIV, linkage to HIV care, and ART initiation.

Despite the strengths of our study, there are important limitations to consider. Notable limitations include ascertainment bias as participants were recruited from health and community organizations and thus were already engaged in HIV care services and as such, do not reflect the experiences of individuals who never started ART or engaged in care. Recall bias is present as the SHAPE survey asked to recall service use, experiences, and recommendations available to them at the time of HIV diagnosis, which may have required participants to recall information from more than 15 years prior. Additionally, the STOP HIV/AIDS initiative is not well defined in its associated services amongst patients, and as a result, the era of HIV care from 2010 onwards may not be conclusively informed by the STOP HIV/AIDS initiative. We recognize that the benefits of the STOP HIV/AIDS initiative extend beyond just PLWH diagnosed after 2010, and truly stratifying our sample by the exposure of this province-wide initiative is limited. Moreover, due to the limited sample size in the final analytic sample, we were unable to look at more granular points in the implementation of the STOP HIV/AIDS initiative.

Future research ought to examine what barriers exist for the implementation of a supportive and holistic care environment in HIV care. Expansion and replication of the integrated HIV and supportive care models is recommended. Subgroup analysis within the SHAPE cohort would aid in understanding population-specific desire and need for supportive services in the provision of HIV care.

## Conclusions

In conclusion, the implementation of a provincial TasP initiative, which included low-barrier testing and treatment as well as enhanced psychosocial services (e.g. peer support, social work services) significantly improved clinical care experiences and health outcomes among PLWH. In combination with a universal treatment strategy, our results suggest that the TasP framework can contribute to population-wide improvements in clinical and therapeutic outcomes and can be best implemented when PLWH have access to psychosocial supports, including peer-support and community-based organizations.

## Data Availability

The British Columbia Center for Excellence in HIV/AIDS (BC-CfE) is prohibited from making individual-level data available publicly due to provisions in our service contracts, institutional policy, and ethical requirements. In order to facilitate research, we make such data available via data access requests. Some BC-CfE data is not available externally due to prohibitions in service contracts with our funders or data providers. Institutional policies stipulate that all external data requests require collaboration with a BC-CfE researcher. For more information or to make a request, please contact Mark Helberg, Senior Director, Internal and External Relations, and Strategic Development: mhelberg@bccfe.ca. The underlying analytical codes are available from the authors on request.

## Abbreviations

ART: Antiretroviral Therapy
BC: British Columbia
STOP: Seek and Treat for Optimal Prevention of HIV/AIDS
VL: Viral Load

## References

[1] Montaner JSG (2011). Treatment as prevention–a double hat-trick. Lancet (London, England).

[2] Montaner JSG, Hogg R, Wood E, Kerr T, Tyndall M, Levy AR (2006). The case for expanding access to highly active antiretroviral therapy to curb the growth of the HIV epidemic. Vol. 368.

[3] Cohen MS, McCauley M, Gamble TR (2012). HIV treatment as prevention and HPTN 052. Curr Opin HIV AIDS.

[4] Rodger AJ, Cambiano V, Phillips AN, Bruun T, Raben D, Lundgren J (2019). Risk of HIV transmission through condomless sex in serodifferent gay couples with the HIV-positive partner taking suppressive antiretroviral therapy (PARTNER): final results of a multicentre, prospective, observational study. Lancet.

[5] Providence Health Care (2010). Seek and Treat: BC to seek and treat most vulnerable HIV patients.

[6] Clarke CM, Cheng T, Reims KG, Steinbock CM, Thumath M, Milligan RS (2016). Implementation of HIV treatment as prevention strategy in 17 Canadian sites: immediate and sustained outcomes from a 35-month Quality Improvement Collaborative. BMJ Qual Saf.

[7] STOP HIV/AIDS Technical Monitoring Committee BC-CfE. HIV Monitoring Quarterly Report for British Columbia: Fourth Quarter 2018. Wools-Kaloustian KK, editor. PLoS ONE. 2018;4^th^. https://stophivaids.ca/qmr/2018-Q4/#/bc.

[8] Nosyk B, Montaner JSG, Colley G, Lima VD, Chan K, Heath K (2014). The cascade of HIV care in British Columbia, Canada, 1996–2011: a population-based retrospective cohort study. Lancet Infect Dis.

[9] Soto TA, Bell J, Pillen MB, For The Hiv/aids Treatment Adherenc HOACSG (2004). Literature on integrated HIV care: a review. AIDS Care..

[10] Olding M, Enns B, Panagiotoglou D, Shoveller J, Harrigan PR, Barrios R (2017). A historical review of HIV prevention and care initiatives in British Columbia, Canada: 1996–2015. J Int AIDS Soc.

[11] Montaner JSG, Lima VD, Harrigan PR, Lourenço L, Yip B, Nosyk B (2014). Expansion of HAART Coverage Is Associated with Sustained Decreases in HIV/AIDS Morbidity, Mortality and HIV Transmission: The “HIV Treatment as Prevention” Experience in a Canadian Setting. PLoS ONE.

[12] British Columbia Centre for Excellence in HIV/AIDS. B.C. launches province-wide expansion of STOP HIV/AIDS program | BC . 2013. Available from: http://www.bccfe.ca/news/releases/bc-launches-province-wide-expansion-stop-hivaids-program [cited Apr 21 2022].

[13] Lima VD, Brumme ZL, Brumme C, Sereda P, Krajden M, Wong J, et al. The impact of treatment as prevention on the HIV epidemic in British Columbia, Canada. Current HIV/AIDS Reports. 2020;17(2):77–87. 10.1007/s11904-020-00482-6 PMC8797149 32124189

[14] Lourenço L, Colley G, Nosyk B, Shopin D, Montaner JSG, Lima VD, et al. High Levels of Heterogeneity in the HIV Cascade of Care across Different Population Subgroups in British Columbia, Canada. Paraskevis D, editor. PLoS One. 2014;9(12):e115277. 10.1371/journal.pone.0115277 PMC4277297 25541682

[15] BC Centre for Excellence in HIV/AIDS (2019). STOP HIV/AIDS® Program Evaluation (SHAPE) Study.

[16] Bever A, Salters K, Tam C, Moore DM, Sereda P, Wang L (2020). Cohort profile: The STOP HIV/AIDS Program Evaluation (SHAPE) study in British Columbia, Canada. BMJ Open.

[17] Government of Canada. Office of Nutrition Policy and Promotion. Canadian Community Health Survey. Cycle 2.2, Nutrition (2004) : Income-related household food security in Canada. Office of Nutrition Policy and Promotion, Health Canada; 2007.

[18] Zhang W, O’Brien N, Forrest JI, Salters KA, Patterson TL, Montaner JSG (2012). Validating a shortened depression scale (10 item CES-D) among HIV-Positive people in British Columbia, Canada. PLoS One..

[19] Radloff LS (1977). The CES-D Scale. Appl Psychol Meas.

[20] Carter AJ, Bourgeois S, O’Brien N, Abelsohn K, Tharao W, Greene S (2013). Women-specific HIV/AIDS services: identifying and defining the components of holistic service delivery for women living with HIV/AIDS. J Int AIDS Soc.

[21] Collins AB, Parashar S, Hogg RS, Fernando S, Worthington C, McDougall P (2017). Integrated HIV care and service engagement among people living with HIV who use drugs in a setting with a community-wide treatment as prevention initiative: a qualitative study in Vancouver, Canada. J Int AIDS Soc.

[22] Sprague C, Simon SE (2014). Understanding HIV care delays in the US South and the role of the social-level in HIV care engagement/retention: a qualitative study. Int J Equity Health.

[23] Wesseling T, Bever A, McLinden T, Wang L, Chau W, Bingham B, et al. Social Support is Associated with a Lower Likelihood of HIV Treatment Interruptions in British Columbia, Canada. In: Canadian Conference on HIV/AIDS Research. Saskatoon; 2019. https://www.cahr-acrv.ca/wp-content/uploads/2019/04/CAHR-2019-Abstract-Book.pdf.

[24] BC Centre for Disease Control (2020). Summary Report Provincial Point of Care Program.

[25] Minichiello A, Swab M, Chongo M, Marshall Z, Gahagan J, Maybank A (2017). HIV Point-of-Care Testing in Canadian Settings: A Scoping Review. Front public Heal.

[26] Office of the Provincial Health Officer (2014). HIV Testing Guidelines for the Province of British Columbia.

[27] Marrazzo JM, Del Rio C, Holtgrave DR, Cohen MS, Kalichman SC, Mayer KH (2014). HIV prevention in clinical care settings: 2014 Recommendations of the International Antiviral Society-USA Panel. JAMA.

[28] Cohen MS, Chen YQ, McCauley M, Gamble T, Hosseinipour MC, Kumarasamy N (2011). Prevention of HIV-1 Infection with Early Antiretroviral Therapy. N Engl J Med.

[29] Gardner EM, McLees MP, Steiner JF, del Rio C, Burman WJ (2011). The Spectrum of Engagement in HIV Care and its Relevance to Test-and-Treat Strategies for Prevention of HIV Infection. Clin Infect Dis.

[30] Hernández-Ramírez RU, Qin L, Lin H, Leyden W, Neugebauer RS, Althoff KN (2019). Association of immunosuppression and HIV viraemia with non-Hodgkin lymphoma risk overall and by subtype in people living with HIV in Canada and the USA: a multicentre cohort study. Lancet HIV.

[31] Lima VD, Eyawo O, Ma H, Lourenço L, Chau W, Hogg RS (2015). The impact of scaling-up combination antiretroviral therapy on patterns of mortality among HIV-positive persons in British Columbia, Canada. J Int AIDS Soc.

[32] Hogg RS, Heath KV, Yip B, Craib KJP, O’Shaughnessy MV, Schechter MT (1998). Improved survival among HIV-infected individuals following initiation of antiretroviral therapy. J Am Med Assoc.

